# New Wave of COVID-19 Vaccine Opinions in the Month the 3rd Booster Dose Arrived

**DOI:** 10.3390/vaccines10060881

**Published:** 2022-05-31

**Authors:** Camelia Delcea, Liviu-Adrian Cotfas, Liliana Crăciun, Anca Gabriela Molănescu

**Affiliations:** 1 Department of Economic Informatics and Cybernetics, Bucharest University of Economic Studies, 010552 Bucharest, Romania; camelia.delcea@softscape.ro; 2 Department of Economics and Economic Policies, Bucharest University of Economic Studies, 010374 Bucharest, Romania; liliana.craciun@softscape.ro (L.C.); gabriela.molanescu@softscape.ro (A.G.M.)

**Keywords:** COVID-19 vaccination, stance analysis, deep learning, opinion mining, natural language processing

## Abstract

Vaccination has been proposed as one of the most effective methods to combat the COVID-19 pandemic. Since the day the first vaccine, with an efficiency of more than 90%, was announced, the entire vaccination process and its possible consequences in large populations have generated a series of discussions on social media. Whereas the opinions triggered by the administration of the initial COVID-19 vaccine doses have been discussed in depth in the scientific literature, the approval of the so-called 3rd booster dose has only been analyzed in country-specific studies, primarily using questionnaires. In this context, the present paper conducts a stance analysis using a transformer-based deep learning model on a dataset containing 3,841,594 tweets in English collected between 12 July 2021 and 11 August 2021 (the month in which the 3rd dose arrived) and compares the opinions (*in favor*, *neutral* and *against*) with the ones extracted at the beginning of the vaccination process. In terms of COVID-19 vaccination hesitance, an analysis based on hashtags, n-grams and latent Dirichlet allocation is performed that highlights the main reasons behind the reluctance to vaccinate. The proposed approach can be useful in the context of the campaigns related to COVID-19 vaccination as it provides insights related to the public opinion and can be useful in creating communication messages to support the vaccination campaign.

## 1. Introduction

COVID-19, the disease that derived from the SARS-CoV-2 novel coronavirus, has been one of the great challenges that the population worldwide has been confronted with since 10 March 2020, when the World Health Organization [1] announced the pandemic. As a response to the mass diffusion of the disease, governments and international organizations recommended or imposed a series of prevention measures, such as physical distancing, mask wearing and lockdown, which have been seen as beneficial or watched with reluctance by the general public [2,3]. The end of 2020 brought new means of fighting the pandemic through the use of vaccines, which have proven to reduce the risk of severe disease, hospitalization or death [4]. As the levels of immunity are highly dependent of the type of vaccine, type of virus variant, time between doses and other human conditions, a 3rd booster dose has been seen as a necessity in the fight against COVID-19 [4,5,6]. In this context, on 12 July 2021, Israel approved the use of a 3rd vaccine dose for the high-risk population, followed in less than one and a half months by the other age categories [7].

Following the announcement made on 9 November 2020 regarding the development of an effective vaccine against the SARS-CoV-2 virus, the general public has started to share their opinions on social media related to their decisions to take or delay the vaccine and the main reasons behind these decisions [8]. As in the case of other vaccine-related situations, Twitter has been one of the preferred platforms for exchanging opinions related to COVID-19 vaccination as it allows users to easily broadcast information using short public messages called tweets [9,10,11]. The platform has been used even by political figures and government officials to address messages to the general public [12].

Regarding the general opinion related to the COVID-19 vaccine expressed on Twitter, the discussions have evolved over time from simple messages, such as “I refuse”, “I don’t”, “Not in my veins” and “Please vaccinate” to more complicated and better-defended arguments for and against vaccination, some of them mentioning and quoting the opinions of well-known physicians and researchers [13,14]. As a result, the analysis of the messages posted on Twitter can bring new insights into the reasons behind people’s decisions to take the COVID-19 vaccine, either the 1st, 2nd or 3rd dose.

In this context, the present paper aims to analyze the messages written in English from Twitter (known as tweets) in the one month period starting from the date on which the 3rd booster dose was approved for administration in Israel with the purpose of extracting the general public opinion regarding the COVID-19 vaccination process. The selected tweets are not necessarily referring to the 3rd dose but rather to the vaccination process in the analyzed period in general, discussing benefits, side effects, decision, news, etc. During the study period, 3,841,594 tweets were posted in the context of the arrival of the 3rd dose that were extracted and divided into three main categories (*in favor*, *neutral* and *against*) through the use of stance analysis. A series of classical machine learning and deep learning classification algorithms were considered for this analysis. The best-performing classifier was chosen based on its performance in connection with five indicators: accuracy, precision, recall, F-score and area under the receiver operating characteristic (ROC) curve (AUC). The results were analyzed by comparing them with previous equally long periods of time characterized by the first vaccine announcement and the first vaccine administration. Additionally, an analysis based on hashtags, n-grams and latent Dirichlet allocation (LDA) was performed on hesitancy tweets to better highlight the main reasons behind the reluctance to vaccinate.

The remainder of the paper is organized as follows: Section 2 presents the attitudes toward the COVID-19 vaccination process as a result of the studies conducted in the scientific literature. Section 3 is dedicated to the process of data collection and the methodology used in this study. Section 4 presents the COVID-19 vaccine stance dataset and classification performance of the considered classical machine learning and deep learning models. Section 5 focuses on the COVID-19 vaccine stance detection, while Section 6 analyzes the COVID-19 vaccine opinions. The paper closes with discussions, limitations and conclusions.

## 2. Literature Review

Paul et al. [15] studied 32,361 UK adults and determined through a survey that 14% of the respondents were unwilling to vaccinate and 22.5% were unsure about this decision. Analyzing in more depth, the authors determined that the major barriers in managing the COVID-19 pandemic are people’s refusal to take the vaccine and negative attitudes toward vaccines. Considering the demographic characteristics of the respondents in the above-mentioned categories (unwilling to vaccinate and unsure about the decision), Paul et al. [15] pointed out that the distrustful attitudes have been encountered more frequently among individuals with ethnic minority backgrounds, lower annual income, less education and less knowledge related to COVID-19 in general and with the government’s actions in particular. Thus, when responding to the negative attitudes toward vaccines, the government messages should be tailored to address the concerns of these specific categories [15].

Bell et al. [16] used both an online cross-sectional survey and semi-structured interviews to determine parents’ and guardians’ opinions related to the acceptability of a COVID-19 vaccine. The study was conducted with 1252 UK respondents. The authors observed that the parents and the guardians were more likely to accept a COVID-19 vaccine for themselves than for their children. The percentage of the respondents refusing the vaccination or unsure but leaning toward no was 9.9%. Even in this case, ethnicity was pointed out as one of the demographic characteristics that correlated with vaccine refusal. The participants’ common concerns related to COVID-19 vaccination were its safety and effectiveness, which were highly questioned due to the newness and the rapid development of the vaccine [16].

Regarding the European Union, a series of studies concerning the COVID-19 vaccination process have been conducted at country level that highlight the differences in attitudes toward vaccination among the considered countries. Soares at al. [17] analyzed the factors associated with COVID-19 vaccine hesitancy in Portugal and reported 65% hesitancy in their study population. The persons in the hesitant category were mainly young individuals who lost income during the pandemic and had low confidence in the vaccine, in the government measures and in the information strategy [17].

In a study focusing on individuals’ fear of the disease or the vaccine, Karlsson et al. [18] showed that in the case of the Finnish, COVID-19 was perceived as a more threatening disease than influenza or measles, and the respondents who believed the disease was a real threat to their health were more prone to accept the vaccine. In the entire sample, approximatively 75% of the respondents reported that they were likely to accept a recommended vaccine [18].

Italian adults’ likelihood of receiving the COVID-19 vaccine was analyzed by Biasio et al. [19], who found a high percentage of the respondents, 91%, were willing to vaccinate, which the authors found reassuring. Di Giuseppe et al. [20] explored vaccine hesitancy in a university population in the south of Italy, and the majority of the participants in the study, 84.1%, were willing to accept a COVID-19 vaccine. Caserotti et al. [21] found that for the Italian residents included in their study, conducted from February to June 2020, the vaccine hesitancy decreased during the lockdown period, and Rapisarda et al. [22] found little or no predictive power of doctors’ sociodemographic characteristics in their recommending the vaccine; those authors found that positive emotions and vaccine confidence were excellent predictors for vaccine recommendation.

An analysis of French opinion related to the COVID-19 vaccine showed that nearly 75% of the respondents were willing to receive the vaccine [23], and a comparable level of vaccine acceptance, 71.2%, was determined that by Schwarzinger et al. [24] in a survey of a working-age population in France. A study on individuals with HIV (human immunodeficiency virus) living in France was conducted by Vallee et al. [25], and 28.7% of the participants (in a sample of 237 respondents) were hesitant about COVID-19 vaccination, mostly due to concerns related to side effects.

For Slovenia, Petravic et al. [26] reported a 59% intention to vaccinate, with the persons willing to receive the COVID-19 vaccine being mostly men, physicians or medical students, older respondents, individuals who vaccinated for influenza and persons who knew other persons hospitalized for COVID-19 or who knew persons who had died due to COVID-19 [26].

The rates of COVID-19 vaccine acceptance have also been determined in a series of studies featuring US respondents. For example, Malik et al. [27] reported a 67% acceptance rate; in that study, the persons unlikely to accept a COVID-19 vaccine were women, young adults and persons with less than a college degree. Reiter et al. [28] obtained a similar acceptance rate, 69%, and found that individuals were more willing to vaccinate if the vaccination was recommended by a health care provider. Latkin et al. [29] reported 59.1% trust in a potential COVID-19 vaccine, and this level was highly associated with individual sociodemographic and behavioral factors. A higher level of acceptance, 96.7%, was reported by Benis et al. [30] in the case of US social media users, whereas Salmon et al. [24], in a study prior to the emergency use authorization, reported only a 50% COVID-19 vaccine acceptance rate.

Borriello et al. [31] and Dodd et al. [32] determined that vaccine uptake in Australia ranged between 76.55% and 86%. Additionally, Dodd et al. [32] found that the reluctance to receive a vaccine in Australian respondents was associated with inadequate health literacy and lower education levels. Alley et al. [33] analyzed whether the willingness to vaccinate against COVID-19 had evolved as the pandemic progressed and found out that in the case of Australians, the willingness to vaccinate had not changed over time. The authors found that women were more likely to be unsure rather than unwilling regarding COVID-19 vaccination than men [33].

Alfageeh et al. [34] investigated the Saudi population’s acceptance of a COVID-19 vaccine and identified willingness among 48% of a sample of 2137 respondents. Participants with a history of vaccine refusal were more likely to refuse the COVID-19 vaccine [34].

COVID-19 vaccine emotions and discussions on Twitter among a US population were analyzed by Karami et al. [35]. The authors studied the period between November 2020 and February 2021 and determined that the negative sentiment declined over time [35]. Similar periods of time were considered by Liu and Liu [36] and Cotfas et al. [13,14] regarding English tweets. Liu and Liu [36] showed that the public sentiment on COVID-19 vaccines varied over time and depended on geographic location, and Cotfas et al. [13,14] underlined that the general public interest in the vaccination process has presented spikes that can be connected with the major events presented in the media in the selected period. Baj-Rogowska [37] used Twitter data to map the vaccine uptake determinants among Polish users and identified six dimensions with influence on COVID-19 vaccine uptake: access, affordability, awareness, acceptance, activation and assurance.

Other studies have referred to but not been limited to: pricing the COVID-19 vaccine [38]; myths and conspiracy theories on vaccines and COVID-19 [39]; the willingness to receive a COVID-19 vaccine with and without the emergency use authorization [40]; vaccine preferences based on efficacy [41]; comparisons among different individual groups regarding vaccine acceptance [42]; the opinions of medical students, nurses and physicians related to COVID-19 vaccination in Colombia [43], Germany [44], Hong Kong [45], Romania [46] and the US [47,48,49]; COVID-19 dynamics after an immunization program in Israel [50]; the protection offered by the Pfizer-BioNTech COVID-19 booster in Israel [7]; the need to address the hesitancy [51]; and a review of vaccine hesitancy during COVID-19 [52].

Based on the papers mentioned above, studies are either using questionnaires or data extracted from social media in order to mine people’s opinions related to COVID-19 vaccination. As the present study aims to extract general opinion at a global level, the second approach is chosen, in accordance with previous studies regarding vaccination analysis from extracted Twitter data [9,53,54,55].

## 3. Methodology

The public opinions related to COVID-19 vaccine are determined using a stance analysis that follows a series of steps discussed below (Section 3.1). Following the stance analysis, the collected tweets are divided into three categories: *in favor*, *neutral* or *against*. The evolution of the tweets in each of the three categories in the selected period are presented through a trend analysis. Then, considering only the tweets in the *against* category, a further analysis is conducted to identify the main discussion topics that determine the general public’s hesitancy towards vaccines (Section 3.2).

### 3.1. COVID-19 Public Opinion

**Figure 1:**
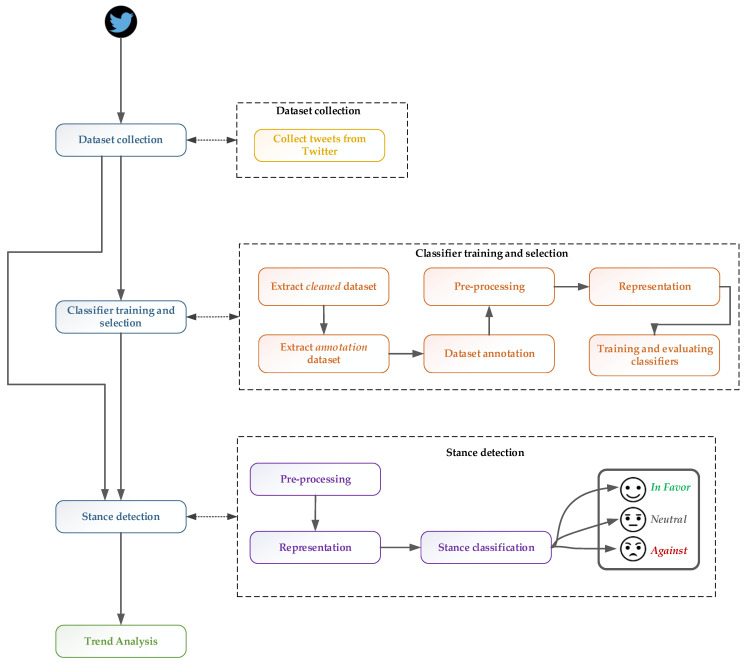
Stance detection steps.

The COVID-19 public opinion was determined by considering the steps presented in Figure 1, namely, data collection, classifier training and selection, stance detection and trend analysis.

#### 3.1.1. Dataset Collection

To extract the tweets in English posted in the month following the arrival of the 3rd booster dose, between 12 July 2021 and 11 August 2021, a search was performed on Twitter using specific keywords related to both COVID-19 (covid19, covid-19, coronavirus, coronaoutbreak, coronaviruspandemic, wuhanvirus, 2019nCoV) and vaccination (vaccine, vaccination, vaccinate, vaccinating, vaccinated). The extracted tweets based on the mentioned keywords comprise the *entire* dataset.

#### 3.1.2. Classifier Training and Selection

From the *entire* dataset, we removed the retweets and duplicated tweets as suggested by Aloufi and Saddik [56] and D’Andrea et al. [9], leaving a *cleaned* dataset that contained unique user-posted tweets. As the number of tweets posted in connection with the COVID-19 vaccine theme is very large, only a small portion of these tweets, 0.53%, were randomly selected for classification into the three categories (*in favor*, *neutral*, or *against*). As the performance of the classifiers could have been adversely affected by training on unbalanced datasets, a balanced annotated dataset was extracted from the annotated dataset that contained an equal number of *in favor*, *neutral* and *against* tweets. This set is further extended by considering the balanced annotated COVID-19 vaccine dataset made freely available by [14], resulting in a combined balanced annotated dataset.

The next step was preprocessing, through which the user mentions, links and email addresses were normalized; the minor spelling mistakes and elongated words were corrected; the emoticons were replaced with their corresponding words; the hashtags were unpacked and all words were set in lowercase. As D’Andrea et al. [9] observed, this step is crucial in achieving results as close as possible to the opinions expressed by users. To perform the preprocessing, ekphrasis library, the Natural Language Toolkit (NLTK) library and the “re” python module [13,57,58] were used.

In the following step, the text is represented as numbers that can be handled by the classification algorithms. In the case of the classical machine learning algorithms, the text was represented using the n-gram language model. Furthermore, to increase the performance of the classification models, term frequency–inverse document frequency (TF–IDF) was employed to reduce the weights associated with the most frequent words, which often carry little information content. For the deep learning classifiers, the pretrained tokenizers provided by the HuggingFace [59] library were used to represent the text through word embeddings.

The last step consisted of the training and evaluation of the classifiers. Various classifiers were considered in accordance with the scientific literature: multinomial naive Bayes (MNB) [60,61], random forest (RF) [62,63], support vector machines (SVMs) [64,65], bidirectional encoder representations from transformers (BERT) [66], the robustly optimized BERT pretraining approach (RoBERTa) [67] and A lite BERT (ALBERT) [68]. The classical machine learning classifiers were implemented using the scikit-learn [69] library, and for the deep learning classifiers, the HuggingFace [59] library was chosen.

The performance of the classifiers was evaluated using five indicators (*Accuracy, Precision, Recall, F-score* and AUC), all of them widely used in the scientific literature related to classification. The calculation formulas for the first four indicators are briefly presented in the following [9]:

(1) $$Accuracy=\frac{TP+TN}{TP+TN+FP+FN}$$

(2) $$Precision=\frac{TP}{TP+FP}$$

(3) $$Recall=\frac{TP}{TP+FN}$$

(4) $$F-score=2\cdot \frac{Precision\;\cdot \;\;Recall}{Precision+Recall}$$

where *TP* is the number of tweets correctly classified as belonging to the analyzed category, *TN* is the number of tweets correctly classified as not belonging to the analyzed category, *FP* represents the number of tweets incorrectly classified as belonging to the analyzed category and *FN* is the number of tweets incorrectly classified as not belonging to the analyzed category.

As a general interpretation, classifiers provide better results with higher indicator values. Based on the evaluation, the best classifier was selected and used for stance detection as follows.

#### 3.1.3. Stance Detection

For the stance detection, preprocessing and representation—similar to what was presented in Section 3.2—were performed on the *entire* dataset.

Then, using the best classifier determined in the previous phase, the stance classification was performed, through which all the extracted tweets were allocated into one of the three categories (*in favor*, *neutral* or *against*).

#### 3.1.4. Trend Analysis

The daily evolution of the number of tweets in each of the three categories (*in favor*, *neutral* or *against*) on both the *cleaned* dataset and the *entire* dataset were analyzed through statistic-specific visual means. A comparison with the evolution of the number of tweets in each of the three categories in other periods considered in the scientific literature was conducted to better observe any changes in the trend corresponding to ongoing vaccination campaigns or to the news related to the 3rd booster dose.

### 3.2. COVID-19 Vaccination Hesitancy Analysis through Hashtags, N-grams and Latent Dirichlet Allocation

Considering only the tweets in the *against* category, further analysis was performed to extract the reasons behind people’s hesitancy related to COVID-19 vaccine administration with the help of hashtags, n-grams and latent Dirichlet allocation (LDA).

#### 3.2.1. Hashtag Analysis

The hashtags associated with the tweets referring to COVID-19 vaccine hesitancy were extracted from the tweets in the *cleaned* and *entire* datasets. Based on their number of appearances, the hashtags with the highest frequency were listed and analyzed.

#### 3.2.2. N-gram Analysis

The n-gram analysis was conducted using the scikit-learn [69] Python library on the *cleaned* set. Even in this case, preprocessing was necessary through which the urls, special characters and stop words provided by the NLTK library [58] were removed, the text was set to lowercase and multiple spaces were reduced to single spaces.

Different types of n-grams, namely, unigrams (1-g), bigrams (2-g) and trigrams (3-g) [70], were considered.

#### 3.2.3. Latent Dirichlet Allocation

Latent Dirichlet allocation (LDA) [71] relies on the bag-of-words paradigm and word-document counts for determining groups that comprise different terms related to a specific topic.

Several steps are needed for LDA that differ from the preprocessing steps we discussed above because the inner workings of LDA vary. First, the punctuations and urls were removed, the text was set in lowercase and the emoticons were replaced with emoji (https://github.com/carpedm20/emoji (accessed on 6 January 2022)). Second, through the NLTK library [58], the text was divided into tokens, and the stop words were removed.

The Gensim (https://radimrehurek.com/gensim (accessed on 6 January 2022)) library [72] was used for topic discovery. Both the original form of tokens and the lemmatized form obtained through the spaCy (https://spacy.io (accessed on 6 January 2022)) library were considered.

The number of topics was determined by analyzing the coherence score, and the selected topics were visualized using the pyLDAvis (https://pyldavis.readthedocs.io (accessed on 6 January 2022)) library. As Chuang et al. [73] suggested, the 30 most salient terms have been provided.

The results were analyzed in the context of the news that appeared during the selected period and by drawing a parallel with the ones provided in [14].

## 4. COVID-19 Vaccine Stance Dataset and Classification Performance

Based on the keywords mentioned in Section 3.1.1, 3,841,594 tweets in English were collected, representing the *entire* dataset. By removing the duplicated tweets and the retweets, a *cleaned* dataset consisting of 876,151 tweets was obtained. The daily evolution of the tweets number in the selected period, 12 July 2021–11 August 2021, is discussed in the following, along with the tweet annotation process and the results regarding the selection of the best classifier.

### 4.1. COVID-19 Vaccine Datasets

The daily distribution of the tweets in both the *cleaned* and the *entire* datasets are presented in Table 1.

**Figure 2:**
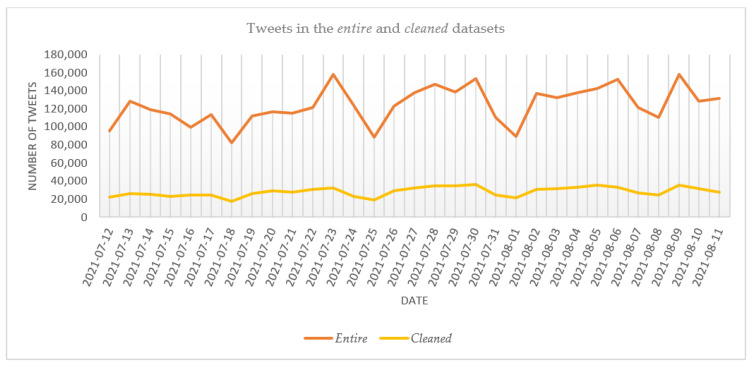
The evolution of the number of tweets in the *entire* and *cleaned* datasets.

The evolution of the number of daily tweets is shown in Figure 2. As the *cleaned* dataset shows, the number of tweets ranged from 17,603 on 18 July 2021 to 36,667 on 30 July 2021, the date with the most tweets. The evolution of the tweets in the *entire* dataset closely matches that in the *cleaned* dataset, with the fewest tweets also published on 18 July 2021, 82,749. The date with the highest overall number of tweets and retweets was 9 August 2021, with 158,312 messages. This date is closely followed by 23 July 2021, with 158,257 messages, and 30 July 2021, with 153,311 messages.

### 4.2. COVID-19 Vaccine Stance Dataset

A total of 4636 tweets were randomly extracted from the *cleaned* dataset, representing approximatively 0.53% of the number of tweets in the dataset. The tweets were manually and individually categorized by three evaluators. The categorization results are presented in Table 2.

During the categorization process, there were no differences in the categorizations of the different evaluators, which could have been because the tweets posted in the analyzed period were very simple, containing warning or encouraging messages, or they were well-stated tweets in which the message was clearly expressed using nearly the full Twitter-allotted message length. A selection of tweets from each of the three categories is presented in Table 3.

A balanced annotated dataset consisting of 1035 tweets was kept for training and evaluating the classifiers’ performance.

The dataset was extended by including a balanced categorized dataset containing 4341 tweets (extracted from a previously classified dataset of 11,187 tweets: 1910 *in favor*, 7830 *neutral* and 1447 *against)* provided in [14].

As a result, the combined balanced categorized dataset comprises 5376 tweets, 1792 tweets in each of the three categories.

### 4.3. COVID-19 Classifiers Performance

For determining the best-performing classifier, both classical machine learning (ML) algorithms and deep learning (DL) algorithms were considered. The best parameters for each model were determined using a grid search approach.

From the first category of machine learning classifiers, multinomial naive Bayes (MNB), random forest (RF) and support vector machine (SVM) were analyzed using different n-gram combinations as features. In the n-gram language model, the text was represented as successions of N consecutive words. The considered n-grams were (1-1)—unigrams; (2-2)—bigrams; (3-3)—trigrams; (1-2)—unigrams and bigrams; (1-3)—unigrams, bigrams, and trigrams; and (2-3)—bigrams and trigrams. Numbers of features limited to 1500, 2000 and 3000 were investigated, as well as the impact of using inverse document frequency (IDF) reweighting. Regarding the stop words, two situations were analyzed, removing the general stop words included in the NLTK library and removing corpus-specific stop words with document frequencies above 0.5, 0.75 and 1.0. Additionally, different values of the parameters specific for each classifier were tested, such as alpha and penalty in the case of the SGDClassifier, equivalent to a linear SVM.

In the case of the MNB classifier, the best results were achieved when representing the text as unigrams and bigrams while limiting the number of features to 3000 and removing corpus-specific keywords using a frequency threshold of 0.75, referred to as ML1 in the following. Additionally, good results were achieved when the ML2 trigrams were included.

The RF classifier also achieved the best results when unigrams and bigrams were used as features and the number of features was limited to 3000. However, in the case of this classifier, better results were obtained when the threshold was set to remove corpus-specific keywords at 0.5 (ML3). Including trigrams as features did not improve the performance of the classifier. This classifier is referred to in the following as ML4.

Good results were also obtained with the SVM classifier when unigrams, bigrams and trigrams were used as features, the total number of features was limited to 3000 and corpus-specific stop words that appeared in more than 75% of the tweets were removed (ML6). The best results for this classifier were achieved when only unigrams and bigrams were employed as features without limiting the total number of features and setting the regularization term to “elasticnet” (noted ML5).

From the deep learning classifiers category, the transformer-based BERT, RoBERTa and ALBERT language models were considered. In the case of the BERT model, the best values for the parameters were determined following the approach recommended by Devlin et al. [66]. The analyzed learning rates were 2 × 10^−5^, 2 × 10^−5^ and 5 × 10^−5^, the batch sizes were 16 and 32 and the numbers of epochs were 2, 3 and 4. Both the version of BERT that ignores the casing of the letters (DL1) and the version that considers case (DL2) were considered. Similar values for the parameters were analyzed for RoBERTa (DL3) and ALBERT(DL4), with the exception of the learning rate, for which 1 × 10^−5^, 2 × 10^−5^ and 3 × 10^−5^ were analyzed as suggested by Liu et al. [67] and Lan et al. [68].

#### 4.3.1. Classifiers’ Performance in Terms of Precision

Precision refers to, for each category, the number of correctly classified tweets among all the tweets that have been classified as belonging to that category. The results of the performance of the ten classifiers in terms of precision are summarized in Table 4. The best results according to this indicator are marked with bold in the table.

Based on the results presented in Table 4, it can be observed that the best classical machine learning classifier was ML3 (69.74%) for detecting the *in favor* tweets and that for the *neutral* and *against* tweets, the best classifier was ML5, at 77.21% and 72.50%, respectively.

In the case of the considered deep learning classifiers, DL3 provided the best results for the three considered categories, scoring 75.65% for *in favor*, 86.62% for *neutral* and 78.89% for *against*.

Overall, the best classifier in terms of precision is DL3, RoBERTa.

#### 4.3.2. Classifiers Performance in Terms of Recall

Recall identifies the proportion of the tweets belonging to a certain category that were correctly classified as belonging to that category. The recall scores for the analyzed classifiers are included in Table 5.

The scores marked with bold in Table 5 show that in the case of *in favor* tweets, the best-performing classifier was ML5 (67.92%), and for *neutral* and *against* tweets, the classifiers offering the highest recall scores were ML6 (74.05%) and ML1 (79.74%).

The best results in the case of the deep learning classifiers were achieved by DL3 for *in favor* (81.16%) and *against* (82.66%) tweets, surpassing the results of the classical machine learning classifiers for all three categories. In the case of *neutral* tweets, the best-performing classifier was DL2, which attained a recall score of 76.61%, also exceeding the scores achieved by the classical machine learning classifiers.

#### 4.3.3. Classifiers Performance in Terms of F-Score

The F-score is computed as a harmonic mean of precision and recall.

As can be observed in Table 6, the best-performing classical machine learning classifier was ML5, achieving F-scores of 68.61 in the case of *in favor* tweets, 75.29 for the *neutral* tweets and 74.59 for the *against* tweets.

From the category of deep learning classifiers, DL3 achieved the highest scores (marked in bold in Table 6) for the three considered categories: 78.26 for *in favor* tweets, 80.69 for *neutral* tweets and 80.68 for *against* tweets. The scores for each category exceed the scores achieved by all the other classifiers, making DL3 (RoBERTa) the overall best-performing classifier.

#### 4.3.4. Classifiers’ Performance in Terms of Accuracy

Accuracy is a statistical measure that highlights the percentage of instances that have been classified correctly. The results of the considered classifiers in terms of accuracy are presented in Table 7.

From Table 7, it can be observed that the best-performing classical machine learning classifier was ML5, which achieved an accuracy score of 72.89%. This score, however, is exceeded by the one attained by the top deep learning classifier, DL3, which has an accuracy of 79.93% (marked in bold in Table 7).

#### 4.3.5. Classifiers Performance in Terms of AUC

The AUC metric measures the area underneath the ROC curve. The ROC curve illustrates the performance of a classifier at different decision thresholds, considering the true positive rate (TPR), also known as recall (Equation (3)), and the false positive rate (*FPR*). The *FPR* can be defined as follows:

(5) $$FPR=\frac{FP}{FP+TN}$$

The AUC ranges between 0% and 100%, with a model providing perfect classification results having an AUC of 100%, while one that outputs only wrong results will have an AUC of 0%. Since AUC is a metric designed for evaluating binary classifiers, in the present paper, a one-vs.-rest approach was used since the number of considered categories is equal to three. The results of the considered classifiers in terms of AUC are presented in Table 8.

As shown in Table 8, the best-performing classical machine learning classifier was ML5 with an AUC of 87.67%. Based on the AUC results, all the deep learning classifiers exceeded the performance of the classical machine learning ones.

The overall best-performing classifier in terms of AUC was DL3, namely the one using the RoBERTa language model, with an AUC of 93.48% (marked in bold in Table 8). Considering the results obtained by each of the ten classifiers for the five performance indicators, it has been determined that RoBERTa (DL3) provided the best performance in almost all situations. Since its performance was exceeded by another classifier only in the case of the recall metric for the *neutral* category. DL3 was used to classify the tweets in the following.

**Figure 3:**
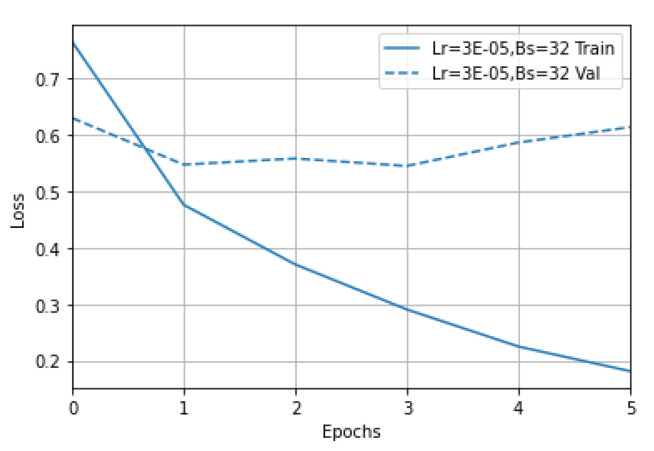
The learning curves for the best-performing classifier.

The learning curves for the best-performing classifier, DL3, are shown in Figure 3, which highlights the evolution of the loss metric on the training and the validation datasets, while the number of epochs increases. As identified through the grid search approach and verified using the learning curves, the best results for RoBERTa were achieved by training for three epochs a model with a learning rate equal to 3 × 10^−5^ (noted with 3E-05 in Figure 3) and using a batch size of 32.

## 5. COVID-19 Vaccine Stance Detection

Based on the stance classification performed with RoBERTa on both the *entire* and *cleaned* datasets, the daily evolution of the number of tweets in each category (*in favor*, *neutral* and *against*) was determined and is discussed in the following.

### 5.1. Stance Detection and Trend Analysis on the Entire Dataset

**Figure 4:**
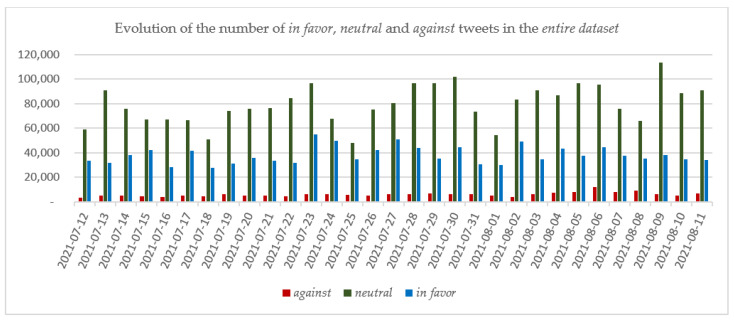
The evolution of the number of *in favor*, *neutral* and *against* tweets in the *entire* dataset.

The evolution of the tweets in the *entire* dataset, for each of the *in favor*, *neutral* and *against* categories, is depicted in Figure 4.

Considering the number of tweets in each category, the most tweets were recorded for the *neutral* category (2,469,434 tweets, representing 64.28% of the *entire* dataset), followed by *in favor* (1,184,223 tweets, 30.83%) and *against* (187,937 tweets, 4.89%). The division of tweets into the three categories on a daily basis showed that the number of *neutral* tweets continued to be the highest in each day of the analyzed period compared with the number of *in favor* and *against* tweets and that on no day did the number of *against* tweets surpass the number of *in favor* tweets (Figure 4).

On 12 July 2021, the date on which the 3rd booster dose was approved for administration in Israel, 95,877 tweets were collected. Based on the RoBERTa classification results, 61.48% of the tweets were in the *neutral* category, 34.79% were *in favor* and 3.73% were in the *against* category. Compared with the average percentages determined for the entire period, it can be stated that the percentage of the *in favor* tweets was higher on the date the 3rd booster dose was approved for administration in Israel than in the analyzed period, while both *neutral* and *against* percentages had lower scores, showing that the announcement regarding the administration of the 3rd dose generated a wave of hope among social network users. As in other cases, it was observed that responses on social networks to current events might lag the event by a day, which was reflected here in that on 13 July 2021, the number of tweets in the *neutral* category increased from 58,947 to 90,721 tweets. Even the number of *against* tweets increased from 3577 to 5404, while the number of *in favor* tweets remained nearly the same.

Over the entire period, there were four days on which the number of tweets exceeded 150,000: July 23 and 30 and August 6 and 9.

On the last day of the analyzed period, 11 August 2021, 131,879 tweets were collected, 5.25% *against*, 68.88% *neutral* and 25.86% *in favor*. Comparing the percentages with the ones recorded for the first day of the analyzed period shows that the percentages for both *against* and *neutral* increased and the percentage *in favor* decreased. Considering the absolute values, the number of *in favor* tweets were nearly the same on the first and last days of the analyzed period, whereas the numbers of *against* and *neutral* tweets on the last day were nearly double the number on the first day. This increase in the number of *against* tweets can be observed throughout the considered period, especially on days near the dates of major events (e.g., July 23 and 30 and August 6 and 9).

Certain tweets have been extensively retweeted. The most retweeted tweet in the *in favor* category, counting 42,728 retweets, is “*I just left the ER. We are officially back to getting crushed by COVID-19. Delta Variant is running rampant and it’s MUCH more transmissible than the original virus. 99% of our ICU admits did NOT receive a vaccine. Virtually ALL of them wish they had*.” As for the other two categories, the most retweeted *neutral* tweet (15,315 retweets) is “*SCOOP: Tennessee Department of Health halts all vaccine outreach to kids—not just for COVID-19, but all diseases—amid pressure from GOP. Staff ordered to remove the agency logo from any documents providing vaccine info to the public, per internal dox. https://t.co/PX0Rvpc6Ot*”, while the most retweeted tweet in the *against* category (3686 retweets) is “*Review of CDC data on COVID-19 deaths in kids showed that zero children have died from COVID-19 in U.S. without pre-existing conditions. Based on the data, mandating a COVID-19 vaccine for all children is unnecessary and unsubstantiated by the science*”.

### 5.2. Stance Detection and Trend Analysis on the Cleaned Dataset

**Figure 5:**
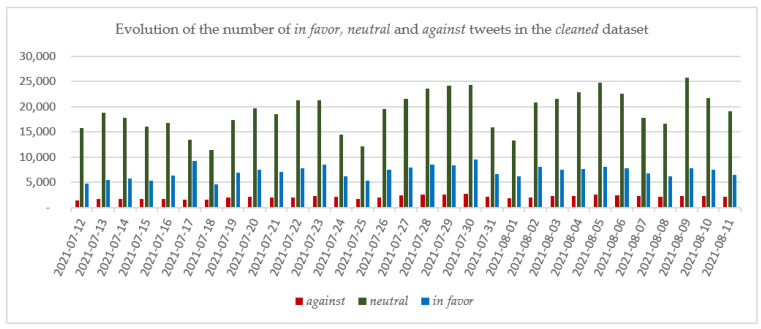
The evolution of the numbers of *in favor*, *neutral* and *against* tweets in the *cleaned* dataset.

In the case of the tweets included in the *cleaned* dataset, it can be observed that the classification into the three categories *in favor*, *neutral* and *against* follows the trend observed in the case of the *entire* dataset but at a smaller scale as only 876,151 tweets are included in the *cleaned* dataset. As in the case of the *entire* dataset, the number of daily tweets in the *neutral* category exceeded the numbers of both *in favor* and *against* tweets, and on no day were there more *against* tweets than *in favor* tweets. The evolution of the number of tweets in each of the considered categories is depicted in Figure 5.

Overall, it can be observed that the percentage of the *against* tweets in the *cleaned* dataset is higher than that in the *entire* dataset (7.44% versus 4.89%) and the percentage of the *in favor* tweets is lower (25.06% versus 30.83%), showing that users who were antivaccination preferred to express their opinions directly, using their own words, rather than sharing the opinions expressed by other persons. There was a higher percentage of *against* tweets in the *cleaned* dataset compared with the *entire* dataset even on the first day of the analyzed period, 6.48% versus 3.73%. On the other hand, the *in favor* tweets had a lower percentage in the *cleaned* dataset compared with the *entire* dataset on 12 July 2021, 21.53% versus 34.79%.

The number of daily tweets was, on average, approximatively 28,263. Several peaks were recorded on the same days as in the *entire* dataset or within 1–3 days around those dates.

Considering the types of vaccines, the most mentioned vaccine was the one produced by Pfizer-BioNTech, totalizing 32,067 tweets (*against*: 2405 tweets, 7.50%; *neutral*: 25,045 tweets, 78.10%; *in favor*: 4617 tweets, 14.40%), followed by Moderna with 14,341 tweets (*against*: 806 tweets, 5.62%; *neutral*: 11,679 tweets, 81.44%; *in favor*: 1856 tweets, 12.94%) and AstraZeneca with 10,383 tweets (*against*: 622 tweets, 5.99%; *neutral*: 8611 tweets, 82.93%; *in favor*: 1150 tweets, 11.08%).

## 6. COVID-19 Vaccine Opinions Analysis

In this section, the COVID-19 vaccine opinions extracted in the month following the arrival of the 3rd dose in Israel (12 July 2021–11 August 2021) are connected with the evolution of people’s opinions regarding COVID-19 vaccination in two other periods, namely, the month following the announcement that a COVID-19 vaccine would be available (9 November 2020–9 December 2020) and the month following the start of the vaccination campaign in the UK (8 December 2020–7 January 2021). The purpose of this analysis was to observe the evolution and structure of the *in favor*, *neutral* and *against* tweets after these three major events, as well as to see whether a certain trend could be identified.

Next, as most of the governments have been interested in counteracting the general public reasons for refusing the COVID-19 vaccination and have tried through specific campaigns to convince people to receive the vaccine, hashtag, n-gram and LDA analysis are performed on the *against* tweets. The results are discussed in comparison with those extracted for the one-month period following the start of the vaccination campaign in UK (8 December 2020–7 January 2021) to identify if certain reasons associated with vaccine refusal remained the same over time.

### 6.1. A Parallel with Other Periods

Two periods that were similar in length but preceded the arrival of the 3rd dose in Israel were selected to observe if there was some change in the structure of *in favor*, *neutral* and *against* tweets in the overall daily number of collected tweets. The two periods were the month following the announcement that a COVID-19 vaccine would be available (9 November 2020–9 December 2020) and the month following the start of the vaccination campaign in the UK (8 December 2020–7 January 2021). The tweets extracted from the two periods and their classifications into the three categories (*in favor*, *neutral* and *against*) were discussed in [13,14]. The tweets in the two periods were selected using the same keywords as in the present paper, which ensured that the comparison had the same foundation. The numbers of *cleaned* tweets extracted from the three periods differed:

- 763,939 tweets between 9 November 2020–9 December 2020 (P1);
- 1,221,694 tweets between 8 December 2020–7 January 2021 (P2);
- 876,151 tweets between 12 July 2021–11 August 2021 (noted P3).

The numbers of daily tweets in the three categories were calculated by dividing the number of daily tweets in each category (*in favor*, *neutral* and *against*) by the total number of daily tweets.

**Figure 6:**
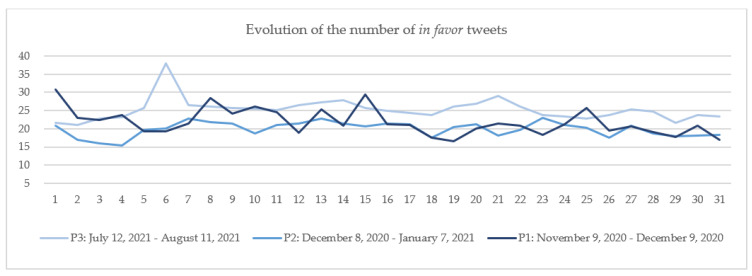
The evolution of the number of *in favor* tweets.

**Figure 7:**
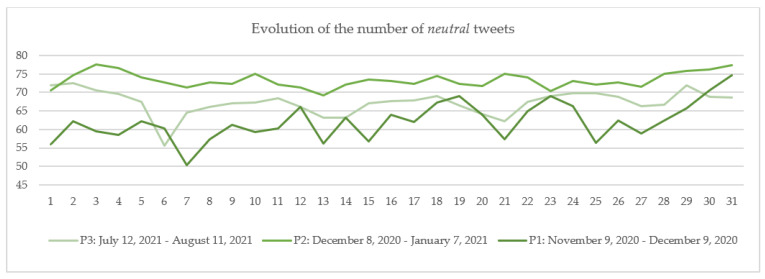
The evolution of the number of *neutral* tweets.

**Figure 8:**
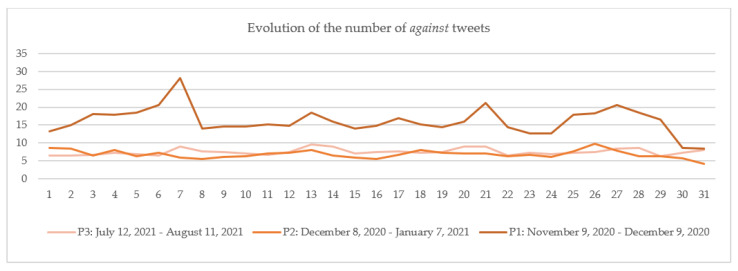
The evolution of the number of *against* tweets.

The results are plotted for each category: *in favor* (Figure 6), *neutral* (Figure 7) and *against* (Figure 8) in each of the three periods: P1 marked with dark color, P2 with medium-light color and P3 with light color. The days considered in the analysis are numbered 1 to 31, 1 being the first day of each period and 31 the last day of that period. If we consider, for example, day 1, Figure 6, Figure 7 and Figure 8 show that the number of tweets *in favor* in P1 is.70%; for *neutral*, it is 55.98%; and for *against*, it is 13.32% (the sum of percentages for the day 1 in P1 is 100%). For P2, the percentages are: *in favor*, 20.90%; *neutral*, 70.53%; and *against*, 8.57%, and for P3, they were: *in favor*, 21.53%; *neutral,* 71.99%; and *against*, 6.48%.

Comparing the results on day 1 of each period, the day on which major news related to COVID-19 vaccination was announced, it can be observed that the highest number of *in favor* tweets in the total number of posted tweets is reported in P1, when the announcement that a vaccine was available was made, followed by P3 when the start of the 3rd dose vaccination in Israel was announced, and last in P2, when the vaccination campaign in UK started (Figure 6). For the entire period included in the study, it can be observed that the number of *in favor* tweets continued to be lower in P2 (with an average of 19.87%) than in P1 (21.81%) and P3 (25.23%). The differences in the percentages of *in favor* tweets among the three periods can be associated with the hope generated by the announcement that a COVID-19 vaccine is available and with the enthusiasm given by a booster dose in the confront with the Delta variant.

In terms of *neutral* tweets, on average, a higher percentage was recorded in P2 (73.31%), followed by P3 (67.26%) and P1 (62.06%) (Figure 7). Considering the trend in Figure 7, it can be observed that on almost every day in P2, the number of tweets marked as *neutral* was greater than on P1 and P3. As the *neutral* category contains mostly news posted by press agencies or by people from all around the world, it was expected that the number of *neutral* tweets would be higher in P2 than in the other two periods because the administration of a new vaccine against a new disease brought to the attention of the public a series of elements related to vaccine characteristics, e.g., distribution, administration, important persons’ opinions or decisions to take the vaccine, etc. On day 1 of the three periods, there were a relatively equal number of *neutral* tweets in P3 and P2 (71.99% vs. 70.54%) and a lower number of *neutral* tweets in P1 (55.98%). In this case, the differences in the percentages among the three periods can be ascribed to the lack of information related to the COVID-19 vaccine on day 1 of P1.

Regarding the numbers of *against* tweets in the analyzed periods (Figure 8), it can be observed that the percentage in P1 (16.13%) is more than double those in P2 (6.82%) and P3 (7.51%). This situation occurs on every day of the selected periods, as reflected in Figure 8. The high difference can be attributed to the uncertainty generated by the creation of a new type of vaccine based on mRNA (messenger ribonucleic acid), which was accompanied in some cases by misinformation [74,75]. With all these, P3 records an overall higher percentage of *against* tweets than P2.

As both P2 and P3 periods mark the start of a vaccination campaign, one might be interested whether there were changes in the discourse conducted by hesitant persons in connection with COVID-19 vaccine. Thus, in the following, a hesitancy analysis is conducted on the *against* tweets in P3, which is discussed in parallel with the results obtained for the *against* tweets in P2, as presented in [14].

### 6.2. COVID-19 Hesitancy Analysis

The COVID-19 tweets marked as *against* in the extracted datasets were analyzed in the following through a hashtag, n-gram and LDA analysis. The purpose of the analysis was to observe the main discussion topics mentioned by the hesitant people regarding their decision not to take the COVID-19 vaccine. The results are discussed in parallel with the hesitancy reasons extracted in [14] for the month following the start of the vaccination campaign in the UK (8 December 2020–7 January 2021). The purpose of this parallel is to see whether the reasons behind the refusal or delay of the COVID-19 vaccination remained the same or changed in the discourse of the hesitant people.

#### 6.2.1. Hashtags Analysis

The hashtags associated with the tweets referring to COVID-19 vaccine hesitancy extracted from the tweets in the *cleaned* and *entire* datasets, and a hierarchy was established based on their number. As some of the hashtags were common to the tweets marked as *in favor*, *neutral* and *against* (e.g., for the *cleaned* dataset: #covid19, 1273 appearances; #vaccine, 938; #covid_19, 938; #coronavirus, 920; #covid, 679; #vaccinated, 358; #vaccination, 320; #covidvaccine, 293; #deltavariant, 210). Only the hesitant-specific top hashtags were retained for the analysis.

Among the top hesitant-specific top hashtags were cases in which, even though the number of tweets for a specific hashtag high, the tweets were merely the same tweet posted multiple times from the same account or from different accounts. We decided to remove these hashtags from the analysis as they contained a single opinion over the anti-vaccine decision and not the points of view of different Twitter members. A selection of the eliminated hashtags from the analysis is provided in Table 9.

After the above-mentioned tweets were eliminated, the top 4 hashtags referring to hesitancy reasons are listed in Table 10 for both the *cleaned* and the *entire* datasets.

Comparing the top 4 hashtags in the two datasets, it can be observed that three of them, namely, #covidiots, #novaccinepassports and #novaccinepassport, are common in both datasets.

Considering the tweets containing #novaccinespassports and #novaccinepassport, it can be observed that, as their name suggests, they are against the use of the COVID-19 vaccination passports. The main hesitancy reasons behind these two hashtags are:

- Side effects, including death (e.g., “*#NoVaccinePassports The MHRA has received 450 UK reports of suspected ADRs to the Pfizer vaccine in which the patient died shortly after vaccination, 960 reports for the AstraZeneca, 6 for the Moderna and 24 where the brand was unspecified. https://t.co/0OixzpqEUs*”;
- Freedom (e.g., “*Keep it voluntary. “Diversity and inclusion” starts here. #NoVaccinePassports https://t.co/UqicjOZnwb”; There are a lot of hypocrites on #MedTwitter and #MedEd who claim to be in favor of reducing inequalities, yet they support vaccine passports that are in complete defiance of WHO’s recommendations. Shame on you. #NoVaccinePassports https://t.co/8v3BiPOCWJ*”).

In the case of #novaccinepasports, on 19 July 2021, a tweet containing the text “*No tests! Only double vaccinated and holding a current Covid 19 passport from September. The end of freedom as you knew it. Complete medical segregation based on your private medical information.That’s not freedom, it’s tyranny. Hold your line. #NoVaccinePassports #COVID19 https://t.co/gRXXZJTB2W*” was retweeted by multiple accounts 549 times. The same situation occurs on 20 July 2021, when the tweet appears 52 times in the *entire* dataset and on 21 July 2021, when it appeared 3 times. Further investigating the retweeting accounts led to the conclusion that the retweets were by real accounts, and thus, the number presented in Table 10 remained 4058.

The hashtag #covidiots usually appears in tweets mentioning the fact that the persons taking the COVID-19 vaccine are not aware of its side effects (e.g., “*Autopsy On Dead Body Of COVID-19 Vaccinated Individual Reveals Spike Proteins In Every Organ—The True Reporter#COVID19 #COVID #Covid1984 #COVIDIOTS #vaccineSideEffects #VaccinePassport #vaccinedeaths #VaccineMandate #FauciLiedPeopleDied #faucigate https://t.co/exrIW1jPDM*”). 

The side effect of the appearance of clot shots in COVID-19 vaccinated people is mentioned in tweets containing #clotshot (e.g., “*STOP the #ClotShot: Do not require health and social care workers to take covid-19 vaccination https://t.co/Lk0Qi0eFKm*”), while other tweets marked with this hashtag highlight the mistrust in the vaccine due to the limited testing (e.g., “*So no clinical trials for any vaccine modifications? 🤯🤨#clotshots gone wild! @RWMaloneMD @BretWeinstein @VaccineTruth2 https://t.co/rj6aRNBM2p*”). The 194 times this hashtag was identified in the *cleaned* dataset (Table 10) was obtained by reducing the number of times the hashtag appeared in the hesitant tweets by 154, as in this case, a tweet posted multiple times on 15 July 2021: “*The heart of COVID-19 is the PCR test without it the CORONA SCAM will die. The Vaccine and lockdowns rely on the faulty PCR TEST to have any validity. Pull the rug on the PCR #CASEDEMIC and find that the emperor has no clothes!! RT #PCRSCAM #convid19 #clotshot https://t.co/ZvH7a8HQqo*”).

The tweets containing #enoughisenough mostly referred to the decisions of various governments to impose restrictions when visiting public places. In this context, the hesitancy reasons for not taking a COVID-19 vaccine are related to mistrust (e.g., “*Protests on France’s national day:Macron is trying to force people to take the experimental #COVID 19 ‘vaccine’. He wants to stop people from even going in shopping centres/malls unless the experimental ‘vaccine’ has been injected into them.(#ImDone #EnoughIsEnough #Corona) https://t.co/icfTcT5eXc*”; “*With Macron in charge France has become a Stalinist police state run by control freaks.From next month only people who have had the #COVID 19 experimental ‘vaccine’ will be allowed to go on trains, buses and in supermarkets.#ImDone#EnoughIsEnough#FightBack#FightBackBetter https://t.co/8eBDJhDTe0*”) and side effects (e.g., “*Search Results from the VAERS Database. 10,991 Covid Vaccine Deaths now in the USA (that’s only 1% of actual reported deaths). How many deaths are acceptable? Why would anyone willing take this? #CovidVaccinesKillAndMaime #EnoughIsEnough https://t.co/3oLpxWmzQG*”).

**Figure 9:**
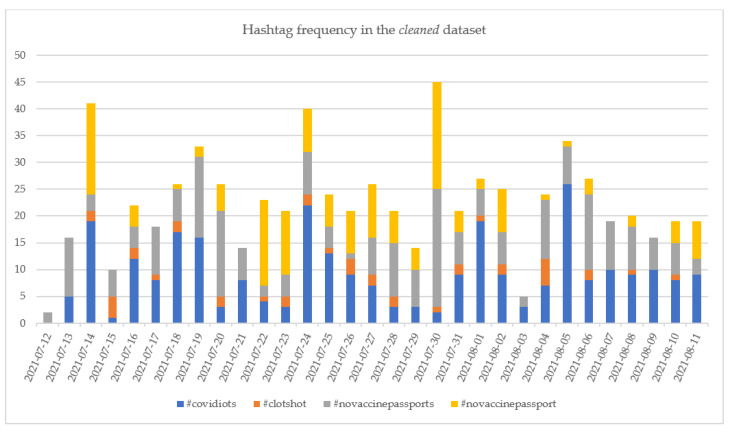
The evolution of the numbers of the top 4 *against* hashtags in the *cleaned* dataset.

**Figure 10:**
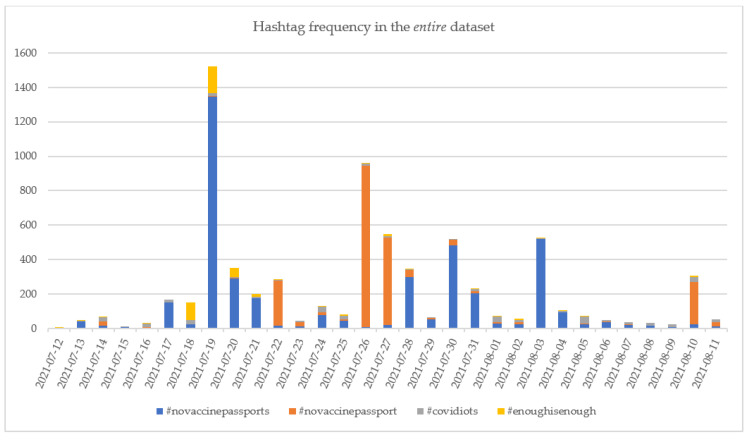
The evolution of the numbers of the top 4 *against* hashtags in the *entire* dataset.

The evolutions of the top 4 hashtags in the *cleaned* and *entire* datasets are presented in Figure 9 and Figure 10.

Regarding the hashtags in the *cleaned* dataset, it can be observed that both #novaccinepassport and #novaccinepassports had peaks on 30 July 2021, recording a total number of 42 tweets. The increased number of tweets with these hashtags compared with the other days in the analyzed period might be because the news regarding the need for the young people to have a vaccine passport in order to attend nightclubs in UK posted on this date (inews.co.uk/news/health/vaccine-passports-young-people-nightclubs-grant-shapps-1129320 (accessed on 27 January 2022)). The #clothshot and #covidiots have been used along the entire period, having few spikes in some days, with no actual connection to a particular news.

Even in the case of the *entire* dataset, the #covidiots has been used along the analyzed period, without being put in connection with particular news. The number of daily tweets containing this hashtag ranged between 0 and 40 tweets in the considered period—Figure 10. Considering other studies in the scientific literature, it has been observed that #covidiots has been reported as one of the hashtags used even at the beginning of the pandemic (between April and December 2020) by the community rejecting or protesting against COVID-19 vaccine, along with other hashtags such as #AntiVacc or #Antivaccine [76]. Regarding the #AntiVacc or #AntiVaccine on the extracted datasets it has been determined that while no #AntiVacc has been reported in the database, the number of tweets containing #AntiVaccine has been rather low, counting only 52 tweets on the *entire* dataset and 23 on the *cleaned* dataset.

The #novaccinepassport recorded a peak in the *entire* dataset on 26 July 2021 when 937 tweets containing this hashtag were published. The increase is justified by the news posted on that day, and most of the tweets discussed the requirement of a vaccine passport for students to return to classes (standard.co.uk/news/uk/downing-street-university-halls-lectures-jabs-covid-vaccine-passport-b947679.html; accessed on 27 January 2022) or to attend sport events (skysports.com/football/news/11661/12364682/premier-league-managers-to-discuss-issue-of-vaccine-passports-amid-government-proposals-around-use-at-sporting-events; accessed on 27 January 2022). Other news posted on 26 July 2021 referred to the need for a vaccination passport in France for dining and travel (npr.org/sections/coronavirus-live-updates/2021/07/26/1020669579/france-new-law-coronavirus-health-pass; accessed on 27 January 2022) or Japan’s introduction of a vaccine passport that exempted the holder from quarantine when traveling to particular countries (japantimes.co.jp/news/2021/07/26/national/japanese-vaccine-passports-start/; accessed on 27 January 2022). As a result of these news stories, the COVID-19 vaccine-hesitant persons expressed their disagreement, which led to the increased use of #novaccinepassport.

Both #enoughisenough and #novaccinepassports peaked on 19 July 2021, encountered in, respectively, 155 and 1346 tweets. The increase in the use of the two hashtags was associated with the declaration of Boris Johnson that admission to mass events would be governed based on vaccine passports starting from September 2021 (cityam.com/vaccine-passport-will-be-compulsory-to-enter-nightclubs-from-september/; accessed on 27 January 2022).

Comparing the top hashtags recorded in the analyzed period with the top hashtags identified for the period between 8 December 2020 and 7 January 2021—the start of the vaccination campaign in the UK—namely, #novaccineforme, #bigpharms, #scamdemic, #billgatesbioterrorist, #covidvaccinesideeffects and #malefertility [14], it can be observed that none of the hashtags have been the same. In contrast, in the analyzed period, the issues reported in the tweets marked with the hashtags listed in Table 10 were related to side effects, freedom and mistrust; the hesitant tweets posted between 8 December 2020–7 January 2021 addressed, in addition to these issues, a broader range of aspects: hiding relevant information, unsafety, inefficiency, existence of alternatives, scam, and moral and religious issues. The reduction in the variety of discussion topics between the two periods could be due to the number of administered COVID-19 vaccine doses worldwide and to the information campaigns promoted in the public space.

#### 6.2.2. N-Grams Analysis

Different n-grams, specifically unigrams, bigrams and trigrams, were extracted, and the top 15 based on their frequency and relevance are presented in Table 11, Table 12 and Table 13. General n-grams not referring to hesitancy reasons or facts (e.g., “covid”, “vaccine”, “vaccinated”, “coronavirus”, “covid19”, “says”) were excluded from the analysis.

Based on the extracted unigrams, presented in Table 11, and on the categories provided in [14], the discussion topics were in the areas of unsafety, side effects, existence of alternatives and hiding relevant information. Comparing the unigrams with the those reported in [14] for the 8 December 2020–7 January 2021 period, it can be observed that some of them have been encountered in both periods: “effects”, “effective”, “risk”, “dangerous”, “death”, “experimental”, and “die”, underlining the fact that the side effects have been a major issue that hinders people from taking the COVID-19 vaccine.

The bigram and trigram analysis (Table 12 and Table 13) revealed that the major discussion topics were unsafety, side effects, mistrust, existence of alternatives, hiding relevant information and scam.

Even in the case of the bigrams and trigrams, it can be observed that some of them remained in the top 15 extracted n-grams in the analyzed period, in contrast with the period 8 December 2020–7 January 2021. The common bigrams for the two periods were: “side effects”, “long term”, “herd immunity”, “immune system”, “big pharma” and “experimental vaccine”, and the common trigram was “long term effects”. The common n-grams show that three of the discussion topics remained common over time, namely side effects, unsafety and the existence of alternatives.

#### 6.2.3. Latent Dirichlet Allocation

**Figure 11:**
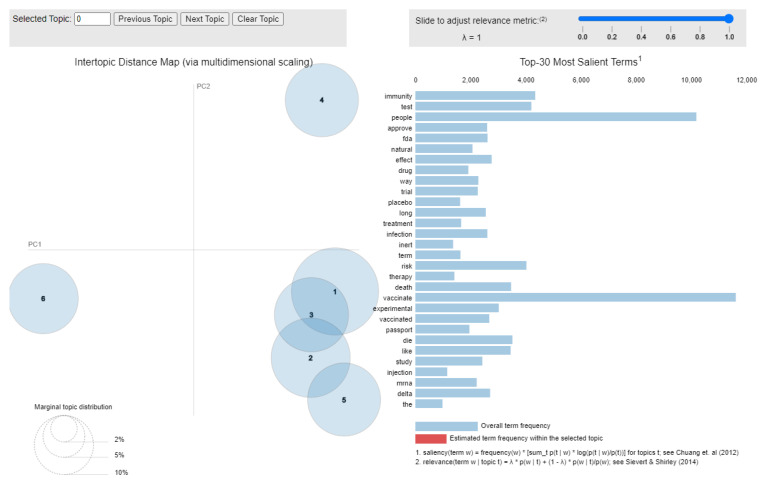
The LDA topics and salient terms.

An LDA analysis was performed of the *against* tweets included in the *cleaned* dataset with the purpose of shaping the discussion topics around the COVID-19 vaccine-hesitant tweeters. The results in terms of discussion topics and included keywords are summarized in Table 14, and the most salient terms are depicted in Figure 11.

Based on the LDA analysis, five main topics could be identified: side effects, existence of alternatives, hiding relevant information, mistrust and scam. Compared with the LDA analysis conducted for the 8 December 2020–7 January 2021 period, there are fewer topics once again as inefficiency and mistrust did not appear in the *against* tweets from the 12 July 2021–11 August 2021 period.

In addition to the general words such as “vaccinate”, “people” and “test”, the most salient words reported in Figure 11 are “immunity”, “risk”, “death” and “die”, once more underlining the focus on the side effects and the existence of alternatives as reasons to reject or postpone the COVID-19 vaccination.

## 7. Discussions and Limitations

By comparing the relative number of tweets in each category (*in favor*, *neutral* and *against*) with an equally large period analyzed in [14], it was observed that the number of *against* tweets was slightly higher in the period considered in this paper than in the period following the start of the vaccination process in the UK, which grounded the need to analyze more in depth the main reasons behind the reluctance to vaccinate in the case of Twitter users. Based on the n-gram, hashtag and LDA analysis, it was observed that the main discussion topics remained the same during the two analyzed periods: most of the discussion topics were in the areas of side effects, the existence of alternatives, hiding relevant information, mistrust and scam. In this context, with the arrival of the 4th dose, it is expected that if no measures are taken by the authorities in charge, the reasons behind vaccination hesitancy will stay the same.

Considering other papers written in the scientific literature, it can be observed that some of the discussion themes in this paper match the themes identified by Küçükali et al. [77] in a study of tweets extracted in the 9 December 2020–8 January 2021 period. Even though the names of the discussion themes are slightly different in the present paper than those in [77], it is possible to establish a correspondence between the names of the discussion themes based on their descriptions, e.g., poor scientific process corresponds to mistrust, and conspiracy theories corresponds to scam. Discussion topics such as side effects, scam, hiding relevant information are common to the ones identified by Karami et al. [35] on a US population for the period between November 2020 and February 2021 and by Herrera-Peco et al. [78] regarding Spanish-written Twitter messages posted between 14 December 2020 and 28 December 2020, again demonstrating that some of the discussion topics remained the same from the start of the vaccination campaign.

This study has limitations that emerge from the process of the identification of the tweets that were included in the analysis, as the selection of the tweets was highly dependent on the keywords used. The classification process also brings some new limitations related to the capacity of the selected classifier to properly identify the tweets from each category given irony is, in most cases, difficult for classifiers to completely understand [10,11]. The social media channel chosen for extracting the messages, namely Twitter, and the considered one-month period represent other limitations of the study. The language of the tweets represents another limitation, along with the period of the analysis. By altering any of the above-mentioned elements, the analysis results could differ slightly. Additionally, we did not consider the geographic locations or the personal characteristics of the individual persons posting the tweets, which should be analyzed in future research.

## 8. Conclusions

This paper analyzed 3,841,594 tweets that were extracted for the 12 July 2021–11 August 2021 period, representing the one-month period starting from the date in which the 3rd booster dose was approved for administration in Israel, for the purpose of analyzing the general opinion regarding the COVID-19 vaccination process. As a result of the analysis, the tweets were divided into three categories: *in favor*, *neutral* and *against* COVID-19 vaccination. As expected, the number of *neutral* tweets exceeded the number of *in favor* and *against* tweets in both the *cleaned* and the *entire* datasets (64.28% *neutral* versus 30.83% *in favor* and 4.89% *against* in the *entire* dataset and 67.50% *neutral* versus 25.06% *in favor* and 7.44% *against* in the *cleaned* dataset). This result is in line with the conclusions of other studies of Twitter using the same keywords for the tweet extraction and featuring other periods since the start of the vaccination process [13,14].

The results of this study are relevant for people interested in observing how the messages related to COVID-19 vaccination posted by Twitter users have evolved in the period following the announcement of the start of the 3rd dose administration in Israel. In addition to the identified distribution of the messages into the three considered categories (*in favor*, *neutral* and *against*), the identified discussion topics related to hesitant tweets can be of interest for the persons directly involved in shaping pro-vaccination campaigns by observing the main reasons of the hesitant people and better addressing them in the future.

This work can be extended by conducting a stance analysis over a longer period in order to better observe the dynamics of the opinions related to COVID-19 vaccination in connection with the variants of COVID-19 (e.g., delta, omicron). Viral slogans and information cascades could also be analyzed to establish a possible correlation with external events such as political health news or upcoming elections. Additionally, an analysis for detecting fake tweets could be performed with the purpose of determining which might be the topics influencing the vaccine-hesitant persons. An analysis could also be conducted of *in favor* tweets to highlight pro-vaccination stances and use them in vaccination campaigns.

## Figures and Tables

**Table 1:** The distribution of the number of tweets published in the analyzed period.

Date		*Entire* Dataset	*Cleaned* Dataset	Date		*Entire* Dataset	*Cleaned* Dataset	Date		*Entire* Dataset	*Cleaned* Dataset
July	12	95,877	21,899	July	23	158,257	32,204	August	3	132,002	31,384
	13	128,208	25,935		24	123,736	22,974		4	137,450	32,835
	14	119,361	25,158		25	88,270	19,193		5	142,740	35,537
	15	114,203	23,085		26	122,915	29,234		6	152,467	32,887
	16	99,482	24,838		27	137,744	32,036		7	121,262	26,787
	17	113,643	24,291		28	147,068	34,692		8	110,653	24,929
	18	82,749	17,603		29	138,891	35,125		9	158,312	35,770
	19	111,832	26,275		30	153,311	36,667		10	128,561	31,463
	20	117,093	29,349		31	110,618	24,883		11	131,879	27,946
	21	114,847	27,641	August	1	89,678	21,433	TOTAL		3,841,594	876,151
	22	121,548	31,068		2	136,937	31,030				

**Table 2:** Statistics for the classified dataset.

Class	*In Favor*	*Neutral*	*Against*	TOTAL
**Number**	1164	3127	345	4636
**Percentage**	25.11%	67.45%	7.44%	100.00%

**Table 3:** Sample tweets.

Stance	Tweet
*in favor*	@Shonib4u @Barbara56532914 @mmaltaisLA @USATODAY Not odd. Medical intervention has only been used for dangerously low oxygen levels. As has been pointed out, 98% of people who Covid-19 survive w/o medical treatment. I understand that 2% is small but chances of vaccine averse events is 0.00001% which is much, much less.
	YES, it’s worse because of selfish people not willing to get Covid-19 vaccine. #GetVaccinated https://t.co/xqo72l6RAC
	Birth control pills have a higher risk of blood clots than the covid-19 vaccine. Go get the vaccine guys
*neutral*	💉🏫 The California State University system announced it will require students, faculty and staff on-campus this fall to be vaccinated against COVID-19. 🏫💉 https://t.co/y6YA2pgpaN
	Stanford University reported at least seven confirmed cases of COVID-19 among fully vaccinated students this week. https://t.co/9R906AQEmU
	United Airlines will require U.S. employees to be vaccinated against COVID-19, joining a growing list of corporations responding to a surge in virus cases. https://t.co/iPjv1UK85o
*against*	More and more story’s like this are being exposed. These injections are pure poison. Autopsy on Dead Body of COVID-19 Vaccinated Individual Reveals Spike Proteins in Every Organ—https://t.co/VSl5l7zXew
	Arrest me if the covid 19 vaccine becomes mandatory because i refuse to inject an aborted fetus into my body, and not know what my future holds. If i have covid i wont be getting tested i refuse to inhale ethylene oxide. do your research or be sheep 🐑👌
	One shot, two shots, three shots… FIVE SHOTS!!! The more shots you take, the more infected you get ! The more infected you get, the more doctors get the HOTS!!! https://t.co/cxTSnjfOqe

**Table 4:** Classifiers performance in terms of precision.

Code	Classifier	Parameters	Class		
			*In Favor*	*Neutral*	*Against*
ML1	**MNB**	n-gram: (1, 2), features: 3000	68.62%	75.50%	68.08%
ML2		n-gram: (1, 3), features: 3000	67.47%	73.55%	67.29%
ML3	**RF**	n-gram: (1, 2), features: all	69.74%	68.72%	65.63%
ML4		n-gram: (1, 3), features: 3000	68.86%	69.60%	65.48%
ML5	**SVM**	n-gram: (1, 2), features: all	69.52%	77.21%	72.50%
ML6		n-gram: (1, 3), features: 3000	67.60%	72.93%	71.87%
DL1	**BERT**	cased: no	73.58%	82.71%	77.90%
DL2		cased: yes	72.77%	79.35%	76.80%
DL3	**RoBERTa**		**75.65%**	**86.62%**	**78.89%**
DL4	**ALBERT**		70.71%	83.45%	73.12%

**Table 5:** Classifiers’ performance in terms of recall.

Code	Classifier	Parameters	Class		
			*In Favor*	*Neutral*	*Against*
ML1	**MNB**	n-gram: (1, 2), features: 3000	62.39%	69.20%	79.74%
ML2		n-gram: (1, 3), features: 3000	59.65%	69.59%	78.57%
ML3	**RF**	n-gram: (1, 2), features: all	53.40%	73.38%	76.28%
ML4		n-gram: (1, 3), features: 3000	55.69%	71.93%	75.67%
ML5	**SVM**	n-gram: (1, 2), features: all	67.92%	73.60%	77.17%
ML6		n-gram: (1, 3), features: 3000	64.90%	74.05%	73.27%
DL1	**BERT**	cased: no	77.58%	75.90%	79.45%
DL2		cased: yes	74.16%	**76.61%**	77.42%
DL3	**RoBERTa**		**81.16%**	75.84%	**82.66%**
DL4	**ALBERT**		74.34%	73.16%	77.67%

**Table 6:** Classifiers’ performance in terms of F-score.

Code	Classifier	Parameters	Class		
			*In Favor*	*Neutral*	*Against*
ML1	**MNB**	n-gram: (1, 2), features: 3000	65.31	72.13	73.39
ML2		n-gram: (1, 3), features: 3000	63.28	71.46	72.44
ML3	**RF**	n-gram: (1, 2), features: all	60.30	70.85	70.46
ML4		n-gram: (1, 3), features: 3000	61.49	70.57	70.05
ML5	**SVM**	n-gram: (1, 2), features: all	68.61	75.29	74.59
ML6		n-gram: (1, 3), features: 3000	66.11	73.45	72.40
DL1	**BERT**	cased: no	75.43	79.01	78.35
DL2		cased: yes	73.32	77.81	76.98
DL3	**RoBERTa**		**78.26**	**80.69**	**80.68**
DL4	**ALBERT**		72.30	77.74	75.07

**Table 7:** Classifiers’ performance in terms of accuracy.

Code	Classifier	Parameters	Accuracy
ML1	**MNB**	n-gram: (1, 2), features: 3000	70.44%
ML2		n-gram: (1, 3), features: 3000	69.27%
ML3	**RF**	n-gram: (1, 2), features: all	67.68%
ML4		n-gram: (1, 3), features: 3000	67.76%
ML5	**SVM**	n-gram: (1, 2), features: all	72.89%
ML6		n-gram: (1, 3), features: 3000	70.73%
DL1	**BERT**	cased: no	77.63%
DL2		cased: yes	76.07%
DL3	**RoBERTa**		**79.93%**
DL4	**ALBERT**		75.05%

**Table 8:** Classifiers’ performance in terms of AUC.

Code	Classifier	Parameters	AUC
ML1	**MNB**	n-gram: (1, 2), features: 3000	86.83%
ML2		n-gram: (1, 3), features: 3000	86.21%
ML3	**RF**	n-gram: (1, 2), features: all	85.19%
ML4		n-gram: (1, 3), features: 3000	84.50%
ML5	**SVM**	n-gram: (1, 2), features: all	87.67%
ML6		n-gram: (1, 3), features: 3000	83.48%
DL1	**BERT**	cased: no	92.07%
DL2		cased: yes	91.17%
DL3	**RoBERTa**		**93.48%**
DL4	**ALBERT**		90.45%

**Table 9:** Hashtags excluded from the analysis.

Hashtag	Occurrences
#pcrscam	202
#casedemic	210
#huntdownmonsters	177
#folksriseup	230

**Table 10:** Top 4 specific hashtags for *against* tweets.

*Cleaned* Dataset		*Entire* Dataset	
#covidiots	282	#novaccinepassports	4058
#novaccinepassports	225	#novaccinepassport	2198
#clotshot	194	#covidiots	433
#novaccinepassport	151	#enoughisenough	392

**Table 11:** Top 15 selected unigrams.

Unigrams	Number of Appearances
immunity	4271
risk	3161
experimental	2907
delta	2898
fda	2704
effective	2500
effects	2214
approved	2207
disease	2103
deaths	2060
flu	1913
death	1767
placebo	1500
die	1341
dangerous	1180

**Table 12:** The top 15 selected bigrams.

Bigrams	Number of Appearances
fully vaccinated	2033
natural immunity	1499
delta variant	1487
side effects	1356
long term	1223
fda approved	983
vaccine passport	954
experimental vaccine	835
immune system	769
placebo vaccine	716
big pharma	712
vaccine passports	659
placebo substance	670
informed consent	604
herd immunity	586

**Table 13:** Top-15 selected trigrams.

Trigrams	Number of Appearances
placebo vaccine passport	700
placebo substance treatment	670
natural immunity vs	515
risk covid 19	467
fully vaccinated people	434
fda approved vaccine	380
covid 19 delta	369
beast natural immunity	349
pfizer biontech covid	347
vaccines worsening clinical	319
trial subjects risk	318
consent disclosure vaccine	316
long term effects	306
let big pharma	295
vaccine recipients severe	270

**Table 14:** The LDA topics, keywords and discussion topics.

Topic Extracted Using LDA	Keywords Included	Discussion Topic
Topic 1	risk, trial, mandate, shot, virus, vaccinate, disease, health, dose, clinical	Side Effects
Topic 2	immunity, vaccinate, way, natural, infection, study, mrna, effective, delta, big	Existence of Alternatives
Topic 3	approve, fda, effect, long, drug, experimental, placebo, treatment, term, passport	Hiding Relevant Information
Topic 4	people, vaccinate, virus, get, vaccinated, die, variant, work, mask, fully	Mistrust
Topic 5	test, know, vaccinate, come, pandemic, stop, corona, pcr, positive, die	Scam
Topic 6	death, like, force, cause, require, life, read, virus, say, rate	Side effects
